# Photochemistry of pyruvic acid is governed by photo-induced intermolecular electron transfer through hydrogen bonds†

**DOI:** 10.1039/d2sc03038a

**Published:** 2022-10-03

**Authors:** Jennifer S. Lewis, Adam P. Gaunt, Arnaud Comment

**Affiliations:** Cancer Research UK Cambridge Institute, University of Cambridge Robinson Way Cambridge CB2 0RE UK arnaud.comment@ge.com; General Electric Healthcare Pollards Wood, Nightingales Lane, Chalfont St Giles HP8 4SP UK

## Abstract

Despite more than 85 years of research, the mechanism behind the photodecarboxylation of pyruvic acid remains elusive. Most studies focused on the gas and liquid phase of diluted solutions of pyruvic acid to understand the impact of sun light on the degradation of this molecule in the atmosphere. By analyzing concentrated supercooled solutions at 77 K, we demonstrate that instead of decarboxylating, the pyruvic acid molecule plays the role of electron donor and transfers an electron to an acceptor molecule that subsequently degrades to form CO_2_. We show that this electron transfer occurs *via* hydrogen bonding and that in aqueous solutions of pyruvic acid, the hydrated form is the electron acceptor. These findings demonstrate that photo-induced electron transfer *via* hydrogen bonding can occur between two simple carboxylic acids and that this mechanism governs the photochemistry of pyruvic acid, providing unexplored alternative pathways for the decarboxylation of photo-inactive molecules.

## Abstract 2

When supercooled pyruvic acid is photo-irradiated, a radical detectable by ESR forms following the transfer of an electron from a molecule in its keto form to a molecule in its hydrated form. The latter subsequently degrades to CO_2_ and acetic acid.

## Graphical abstract

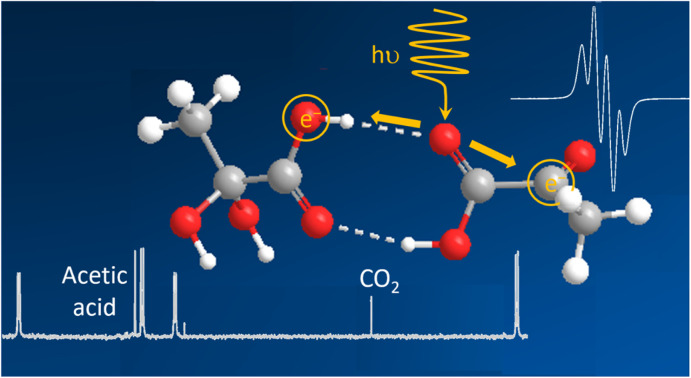

## Introduction

The photochemistry of pyruvic acid (PA) has been the subject of numerous studies, some dating back more than 85 years.^1^ All of them have reported a decarboxylation process upon irradiating this simple alpha-keto acid with ultraviolet (UV) light, leading to the production of CO_2_. It has also been clearly established that ^3^[PA]*, the triplet excited state of PA formed when the photo-excited singlet state undergoes intersystem crossing (ISC), is the reactive species initiating the production of carbon dioxide.^2^ However, the fundamental process by which the excited electron separates from ^3^[PA]* and drives the observed photochemical reactions has not been clearly revealed and continues to be the subject of heated debates.^3^ The main reason behind the difficulties in identifying this mechanism is common to many photochemical processes: the radicals created upon photo-irradiation are so short lived that chemical analyses can often only be performed on post-reaction secondary byproducts, leading to speculative hypotheses on the primary reaction mechanisms. This general limitation, together with the variety of experimental conditions that were used in previous studies of photo-irradiated PA, has led to the identification of several different secondary byproducts^4^ and is the cause for the many unsuccessful attempts to provide a complete and unified description of the photochemistry of PA.

Most studies focusing on the photochemistry of PA are related to atmospheric sciences, where PA breaks down under exposure to sunlight.^5–8^ They were conducted either in diluted aqueous liquid-state solutions or in the gaseous phase.^9,10^ Because such experiments are usually performed at low PA concentrations, they are highly susceptible to the presence of impurities in the starting compounds. In addition, since secondary reactions with neighboring molecules can readily occur in gases and liquids, unravelling the photochemical pathways by correlating the byproducts identified in these studies with the initial photo-dissociation steps is challenging.

In the present study, instead of focusing on the gas or liquid phase, we propose to photo-irradiate PA in the amorphous state, at 77 K, following flash freezing of neat and aqueous solutions of PA into liquid nitrogen to form supercooled liquids.^11^ In this state, the frozen solutions still have liquid-like properties, in particular the absence of long-range order,^12,13^ but certain molecular motions are restricted,^13,14^ hence limiting the creation of termination or recombination products. This low-temperature approach also allows direct observation, *via* electron spin resonance (ESR), of the intermediates created upon photo-irradiation which are stable at 77 K. Combining this method with selective ^13^C isotope labeling and subsequent liquid-state nuclear magnetic resonance (NMR) analyses of the melted photo-irradiated supercooled solutions, provides unique insights into the mechanisms behind the decarboxylation process that has been so often reported in samples containing PA molecules.

## Results and discussion

### Nature and concentration of photo-generated radical

A strong X-band ESR signal has previously been observed in photo-irradiated supercooled neat PA at 77 K.^15^ The signal was assigned to a lactyl radical with the unpaired electron localized around the C2 carbon, as demonstrated using ^13^C-labeling.^16^ It was also shown that the same radical can be photo-induced in various supercooled aqueous solutions containing PA together with photo-inactive molecules.^17^ This has been confirmed in the present study by measuring a photo-irradiated supercooled 300 mM aqueous PA solution at 77 K by X-band ESR (Fig. S1†). Besides the sharp line observed around 340 mT, no other ESR signal could be detected. These results clearly show that whether PA is neat or dissolved in H_2_O, the low-temperature ESR signal is from a single unpaired spin (*S* = ½) rather than the biradical (*S* = 1) previously reported by Guzman *et al.*,^18^ emphasizing the unresolved fate of the second photo-generated unpaired electron.

In the presence of H_2_O, PA partially converts to its hydrated form (PA^H^), a reversible transformation that is highly dependent on pH.^19^ Although PA^H^ is photo-inactive, previous studies suggested that PA^H^ might play an important role in the photochemistry of PA.^20^ PA and PA^H^ cannot however, be independently ^13^C-labeled in solution because their interconversion will lead to ^13^C exchange between the two species. We hence propose to use lactic acid (LA) as a surrogate for PA^H^ to assess the role of the hydrate in the photochemistry of PA. LA has a very similar structure to PA^H^, the only difference being that LA has only one hydroxyl group on the C2 carbon, and it is also photo-inactive. In the absence of the lactate dehydrogenase (LDH) enzyme, LA cannot be converted to PA in solution. It is therefore possible to ^13^C-label LA and PA independently. LA can easily be distinguished from PA as well as its hydrate (PA^H^) form in ^13^C NMR spectra. In addition, like PA, LA can also form a supercooled liquid.^13^

**Fig. 1:**
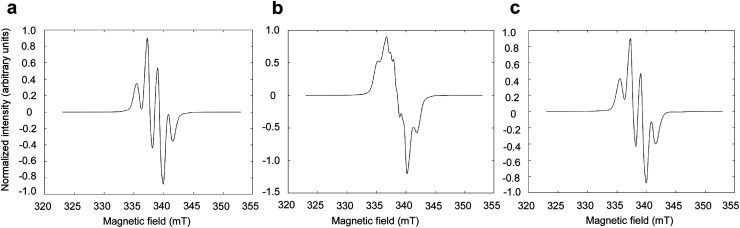
X-band ESR spectra of photo-irradiated supercooled aqueous PA and LA solutions measured at 77 K. (a) PA (300 mM) and LA (8.8 M) in H_2_O. (b) [1-^13^C]PA (300 mM) and LA (8.8 M) in H_2_O. (c) PA (300 mM) and [1-^13^C]LA (8.8 M) in H_2_O.

To assess the effect of the presence of LA on the radical formation in photo-irradiated supercooled aqueous solutions of PA, PA was dissolved at 300 mM in an 8.8 M aqueous LA solution and was flash frozen in liquid nitrogen. The X-band ESR spectrum observed at 77 K is identical to the spectrum previously observed in photo-irradiated supercooled PA,^15^ with a characteristic hyperfine coupling to the 3 methyl protons of PA (Fig. 1A). When natural isotopic abundance PA is replaced by [1-^13^C]PA, the unpaired electron localized on the C2 is affected by the hyperfine coupling to the nuclear spin of the carboxyl ^13^C (Fig. 1B), as previously observed by Eichhorn *et al.*^15^ However, replacing natural isotopic abundance LA by [1-^13^C]LA has no effect on the ESR spectrum (Fig. 1C), confirming that the observed unpaired electron spin is not localized on the LA molecule and that the presence of LA does not alter the nature of the photo-induced radical formed from the reactive triplet state ^3^[PA]*.

**Fig. 2:**
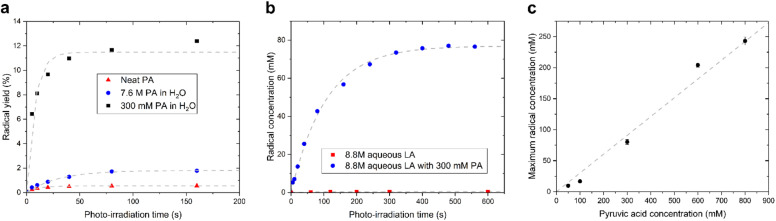
(a) Evolution of radical yield (proportion of radical per PA molecule) as a function of photo-irradiation time in an amorphous supercooled sample containing neat PA (triangles), 300 mM aqueous PA solution (squares), and 7.6 M aqueous PA solution (circles). The light grey dashed curves correspond to the best fits to a simple exponential function with a single time constant. (b) Evolution of radical concentration as a function of photo-irradiation time in amorphous supercooled samples of 8.8 M aqueous LA solution containing either no PA (squares) or 300 mM PA (circles). In the absence of PA, no radical is formed, demonstrating that LA is photo-inactive. The light grey dashed curves correspond to the best fit to a simple exponential function with a single time constant. (c) Maximum radical concentration as a function of PA concentration in amorphous supercooled 8.8 M aqueous LA solution. The grey dashed line is a linear regression (*R*^2^ = 0.97).

We observed that the evolution of the radical concentration and the radical yield (proportion of radical per PA molecule), as a function of the photo-irradiation time reaches a plateau for all supercooled liquids containing PA (Fig. 2). This saturation, which was previously reported in neat and diluted PA samples,^15,17^ indicates that there is a limiting factor in the radical formation. The maximum achievable radical concentration is linearly dependent on the concentration of dissolved PA, up to about 1 M in aqueous solutions (Fig. 2C), but it is however largely below the concentration of PA itself, meaning that the maximum achievable radical concentration is not restricted by the availability of the PA molecule. The radical concentration must therefore be limited by the number of molecules or atoms that can accept the undetected second unpaired electron and it is likely that another molecule besides the one measured by ESR is involved in the mechanism. This molecule can neither be H_2_O since it is highly abundant in all diluted samples, nor oxygen since sparging the solutions with argon gas prior to flash freezing had no impact on the radical concentration (Fig. S2†). Guzman *et al.* speculated that, because PA is known to form dimers, one of the PA molecules plays the role of acceptor.^18^ This interpretation cannot, however, explain the saturation in radical formation observed in neat PA as a function of photo-irradiation time since PA is far from being depleted after 3 min of irradiation and the efficiency by which radicals are created (*i.e.* the yield) in fact increases as the PA concentration decreases (Fig. 2a). Griffith *et al.* hypothesized that ^3^[PA]* could abstract a proton from a neighboring PA^H^, leading to its decarboxylation and the formation of a hydrated acetyl radical.^20^ However, this proposed mechanism is not consistent with the reported ESR results since the acetyl radical should be readily detectable at 77 K.^21,22^

To identify the acceptor molecule and unravel the first photo-dissociation steps, we investigated the reaction products obtained after dissolving the photo-irradiated glassy supercooled ^13^C-labeled PA and LA samples by liquid-state ^13^C NMR.

### ^13^C NMR analyses of photo-induced byproducts

**Fig. 3:**
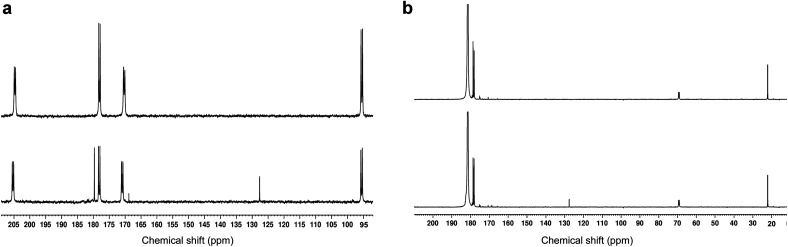
Room-temperature liquid-state ^13^C NMR spectra of dissolved, photo-irradiated and non-photo-irradiated glassy supercooled PA samples. The bottom and top spectrum in each panel corresponds to photo-irradiated and non-photo-irradiated sample respectively. (a) Glassy supercooled 300 mM aqueous [1,2-^13^C_2_]PA sample (24 μL) dissolved in 500 μL D_2_O. Both C1 and C2 keto doublets (170.9 ppm, and 205.3 ppm) along with their respective hydrated form (178.1 ppm, and 95.2 ppm) are visible. Two breakdown products appear on the photo-irradiated spectrum: CO_2_ at 127.6 ppm, and [1-^13^C]acetic acid at 179.7 ppm. A small but significant singlet peak was observed at 169 ppm in the photo-irradiated sample. This peak was not observed in samples prepared with [2-^13^C]PA (see Fig. S3†) and is therefore likely a byproduct from a ^13^C-labeled photosensitive impurity present in [1,2-^13^C_2_]PA; (b) glassy supercooled 300 mM PA in 8.8 M aqueous [1-^13^C]LA sample (24 μL) dissolved in 500 μL D_2_O. The C1 peak of LA was observed at 181.5 ppm along with its two natural abundance ^13^C peaks (C2 at 69.1 ppm, and C3 at 22 ppm). A set of lactic acid impurities previously reported were also detected between 170 and 180 ppm.^23^ A peak corresponding to CO_2_ (127.6 ppm) was only observed in the photo-irradiated sample.

As expected from the previously reported results for [U-^13^C_3_]PA samples,^15^^13^CO_2_ and [1-^13^C]acetic acid peaks were detected in the ^13^C NMR spectra recorded in dissolved photo-irradiated glassy supercooled 300 mM aqueous [1,2-^13^C]PA samples (Fig. 3A). The presence of both acetic acid and CO_2_ as reaction products of photo-irradiated PA has led to many speculations and debates on the photochemistry of PA. This is because their formation from the lactyl radical cannot be easily explained. The fact that only two photolytic breakdown products are formed, and no other termination products are observed, highlights how well controlled the reaction mechanism induced by photo-irradiation is in these experiments.

**Fig. 4:**
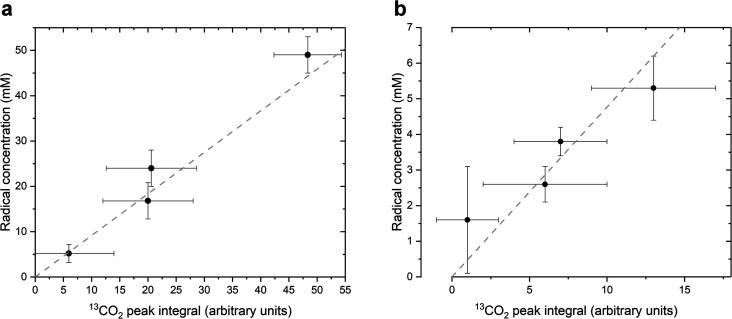
Relationship between the radical concentration measured by ESR at 77 K and the CO_2_ peak integral measured by liquid-state ^13^C NMR in photo-irradiated supercooled PA samples. (a) Photo-irradiated 300 mM PA and 8.8 M [1-^13^C]LA sample measured at different irradiation times (2 s, 5 s 10 s, and 15 s). The grey dashed line is a linear regression (*R*^2^ = 0.99). (b) Photo-irradiated neat PA sample measured at different irradiation times (2 s, 5 s, 10 s, and 15 s). The grey dashed line is a linear regression (*R*^2^ = 0.98).

As stated above, our strategy was to use LA as a surrogate for PA^H^, and by dissolving supercooled photo-irradiated samples in D_2_O we discovered that the liquid-state ^13^C NMR spectra of 8.8 M LA solution containing 300 mM PA exhibits a ^13^CO_2_ peak in samples prepared with [1-^13^C]LA (Fig. 3b) as well as a greater radical concentration (Fig. 4). This result unquestionably demonstrates that the photo-inactive LA molecule undergoes a decarboxylation process upon light irradiation, and that to do so, an unpaired electron has to be transferred from the photo-excited triplet state of PA to LA. In other words, LA plays the role of acceptor for the excited electron of ^3^[PA]*. To confirm that the creation of each unpaired electron detected at 77 K by ESR is associated with the transfer of another unpaired electron which triggers a subsequent decarboxylation on the acceptor molecule, we measured both the concentration of radicals by ESR and the concentration of ^13^CO_2_ by ^13^C NMR in the same photo-irradiated [1-^13^C]LA samples (Fig. 4a). The correlation was clearly linear up to the solubility threshold of CO_2_ in H_2_O, namely a few tens of mM.^24^ A similar linear correlation was obtained between the ESR signal intensity and the ^13^CO_2_ NMR signal in photo-irradiated neat [1-^13^C]PA samples (Fig. 4b), which indicates that [1-^13^C]PA^H^ must play the role of electron acceptor in the absence of LA. This is further evidenced by examining the PA^H^-to-PA ratio in neat and diluted aqueous PA samples before photo-irradiation (Table 1), since this ratio increases with dilution, the concentration of electron acceptor should increase and lead to a higher radical yield. This is indeed in agreement with the data shown in Fig. 2a. It is known that the PA^H^-to-PA ratio is affected by pH.^25^ In more diluted PA samples, the pH and therefore the PA^H^-to-PA will drop, but even in a 10 mM PA sample the pH is low enough (pH = 2) to form a substantial PA^H^ concentration (PA^H^-to-PA ratio of 20%; see Table 1). Therefore, our findings can be extrapolated to the more diluted samples that have been the subject of previous studies.^25,26^

**Table 1:** Ratio between hydrate and keto form of PA measured by ^13^C NMR in different PA solutions prior to photo-irradiation

Solution composition	PA^H^-to-PA ratio (%)
Neat [1-^13^C]PA	7 ± 1
7.6 M [1-^13^C]PA in H_2_O	16 ± 2
300 mM [1-^13^C]PA in H_2_O	38 ± 4
10 mM [1-^13^C]PA in H_2_O	20 ± 2
300 mM [1-^13^C]PA in ethanol	60 ± 6
300 mM [1-^13^C]PA in hexane	0

### Intermolecular electron transfer mechanism

**Fig. 5:**
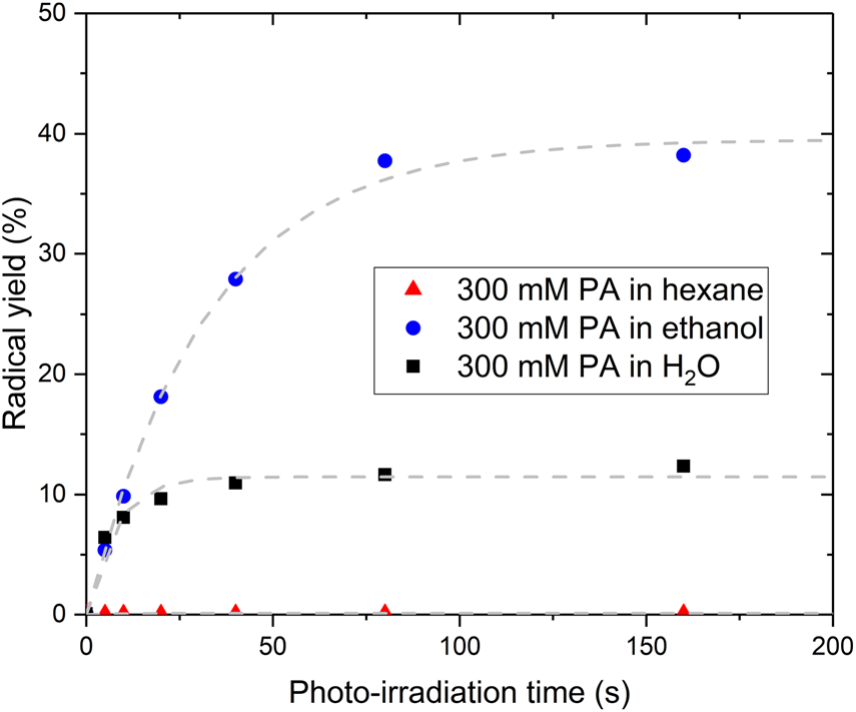
Radical yield as a function of photo-irradiation time for PA samples prepared in different solvents. Evolution of the radical concentration at 77 K as a function of photo-irradiation time in amorphous supercooled 300 mM PA samples prepared in hexane (triangles), ethanol (circles), or H_2_O (squares). The light grey dashed curves correspond to the best fit to a simple exponential function with a single time constant.

The fact that PA itself cannot be the acceptor molecule corroborates previous studies reporting that photo-irradiation of PA dissolved in non-aqueous solvents such as methanol or chloroform resulted in the production of little to no CO_2_.^7^ To further investigate the mechanism behind the intermolecular electron transfer from PA, we replaced H_2_O by hexane or ethanol. No radical could be photo-induced in the sample prepared with hexane while the sample prepared with ethanol exhibits a larger radical yield than the sample prepared with H_2_O (Fig. 5). The ^13^C NMR spectrum recorded post irradiation in the sample prepared with hexane confirmed that no CO_2_ had been produced (Fig. S4†). A ^13^C NMR analysis of the solution before photo-irradiation also confirmed the absence of PA^H^ in this sample. However, the PA^H^-to-PA ratio was 3 times larger in the sample prepared with ethanol than in the sample prepared with H_2_O (Table 1). This partly explains the large radical concentration detected in the sample prepared with ethanol. A complete study of the effect of non-aqueous solvents on the photochemistry of PA is beyond the scope of this paper, but a fundamental difference between hexane and ethanol is that hexane does not contain any hydroxyl group and therefore cannot form any hydrogen bonds with PA. Hydrogen bonds are favored in supercooled aqueous solutions,^27–29^ notably in supercooled aqueous solutions of PA, in which PA-PA or PA-PA^H^ dimers connected through hydrogen bonds can be expected to form.^18,30–33^ The existence of electron transfer between molecules containing carboxylic groups *via* hydrogen bonds has previously been demonstrated.^34^ Together with the evidence of an intermolecular electron transfer from PA, the observation that the photo-irradiation of PA dissolved in hexane does not create any radical leads to the conclusion that the transfer of the excited electron from ^3^[PA]* must occur *via* an intermolecular bond formed by a hydroxyl group, namely a hydrogen bond.

### Limiting factor in radical formation and PA reformation

**Scheme 1:**
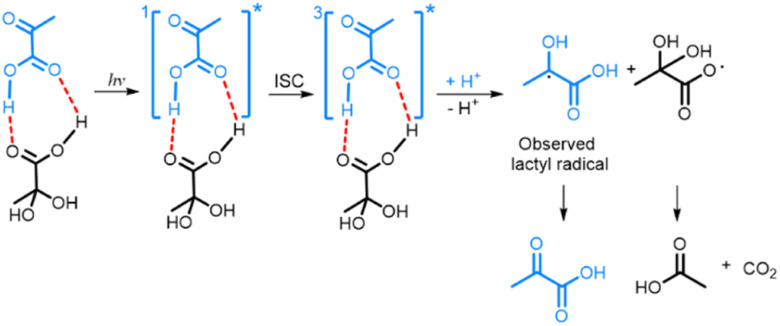
Photo-decarboxylation of PA-PA^H^ pair by UV-vis light. Dashed lines represent the hydrogen bonds between the PA and PA^H^. ISC represents the intersystem crossing mechanism by which the triplet state ^3^[PA]* is created.^8,18,38^

The fact that PA^H^ is the electron acceptor in photo-irradiated supercooled aqueous PA solutions explains the plateau in radical concentration observed beyond a certain photo-irradiation time: the pool size of PA^H^ in the amorphous supercooled samples is finite, which limits the number of PA-PA^H^ dimers and hence the number of radicals. When PA is dissolved in a LA solution, PA forms hydrogen bonds with LA, which plays the role of electron acceptor, and the maximum amount of radicals that can be created is essentially limited by the number of PA molecules that are free to form such bonds. Having shown that the creation of the lactyl radical from PA is associated with the subsequent formation of CO_2_ and acetic acid from PA^H^, it is clear that the lactyl radical has to reform PA when the supercooled solution is warmed up since no other byproduct is detected. It was previously shown by ESR that the lactyl radical disappears around 190 K,^35^ and several mechanisms explaining the recycling of PA have been proposed.^20,36^ Although our data confirms this reformation, it does not allow us to discriminate one or the other mechanism. From the results presented in this paper, we can deduce the likely mechanism resulting from the photo-induced decarboxylation of PA (Scheme 1). Since we could not detect the ESR signal of the radical formed on PA^H^, we concluded that it is not stable at 77 K and that CO_2_ and acetic acid are most likely already formed at this temperature.^8,18,37,38^

## Conclusions

In this study, we demonstrated that the photochemistry of PA is driven by the formation of hydrogen bonds with a molecule that can play the role of an acceptor in an electron transfer from the excited triplet state of PA. We showed that in supercooled aqueous solutions of PA, the hydrated form of PA is the electron acceptor and forms acetic acid in addition to CO_2_. We also proved that the keto form of PA itself does not decarboxylate and that the absence of an electron acceptor and the ability to form hydrogen bonds in the solution prevents the formation of radicals. Although Griffith *et al.* had already proposed a mechanism involving PA^H^,^20^ neither the reported acetyl radical nor LA product were observed in the present study, though it must be noted that the system in which they carried out their experiments was not the same as the one described here. In demonstrating that an electron can be transferred *via* hydrogen bonding from the photoactivated triplet state of PA to other molecules, regardless of their photoactivity, we show that decarboxylation is possible with molecules which may not have been previously considered to add to atmospheric CO_2_. More generally, this demonstrates that intermolecular photo-induced electron transfer (PET), a central photochemical mechanism usually associated with molecules containing aromatic rings,^39,40^ can occur between two simple carboxylic acids.

We proposed using selective isotopic labeling of precursor molecules together with a combination of low-temperature ESR analyses of intermediates and ^13^C NMR analyses of byproducts to unravel the photochemistry of the photosensitive PA molecule in various solutions. The photo-irradiation was performed on supercooled liquids at 77 K following flash freezing of the solutions in liquid nitrogen, providing a snapshot of intermolecular interactions that take place in room-temperature liquid solutions. This has led to the confirmation of photo-induced products from PA as being CO_2_ and acetic acid only. With the methods used we have been able to confirm that the mechanism for this occurrence is due to an electron transfer *via* a hydrogen bond, not a hydrogen abstraction as previously described.^20^ This approach could be used as a general method to study the formation of hydrogen bonds and dimers in the amorphous state of other types of solutions, a state of matter that is still not completely understood.^11,12,41,42^ The results presented herein provide evidence required to fully explain the much debated mechanism of PA photodecarboxylation.

## Materials and methods

### Sample preparation

Unless specified otherwise, all chemicals used in this study were purchased from Sigma Aldrich, Gillingham, UK. All pyruvic acid (PA) samples were prepared from neat PA, using either natural isotopic abundance PA (99% purity) or PA ^13^C-labeled in position C1 (99% ^13^C [1-^13^C]PA, 95% purity), C2 (99% [2-^13^C]PA; 99% purity), or both (99% [1,2-^13^C]PA; 99% purity). 300 mM PA samples were prepared by dissolving neat PA in distilled H_2_O, neat hexane (99% purity) or neat ethanol (99% purity), or in an 80 : 20 (w/w) lactic acid : H_2_O mixture using either natural isotopic abundance lactic acid (LA) solution (90% in H_2_O) or [1-^13^C]LA (99% ^13^C; 98% purity) dissolved in H_2_O to give a 8.8 M LA concentration.

To remove dissolved oxygen from selected samples, a sparging procedure with argon gas was used. Argon gas was bubbled for 30 minutes through a capillary tube into the liquid sample (*e.g.* neat pyruvic acid) located in a round bottom flask.

Frozen samples in the form of beads were produced by flash freezing droplets (4 × 6 μL) of PA sample in liquid nitrogen. Photo-irradiation was performed by placing the beads in the 5 mm outer diameter (OD) tail of a 150 mL quartz dewar (Suprasil Dewar Flask type WG-850-Q, Wilmad, USA) prefilled with liquid nitrogen prior to shining ultraviolet-visible (UV-vis) light using a lightguide connected to a Bluewave 200 broadband source (Dymax, Wiesbaden, Germany) with an output power density of 40 W cm^2^ distributed over a UV-vis spectrum of 280–450 nm. Each 6 μL bead was photo-irradiated individually for a predetermined time.

### X-band ESR measurements at 77 K

ESR measurements of individual beads placed in the 5 mm OD tail of a 150 mL quartz prefilled with liquid nitrogen were carried out using a X-band benchtop spectrometer (Miniscope 400, Magnettech, Berlin, Germany). The radical concentration was determined by comparing the double integral of the ESR spectrum to a predefined calibration curve, created from a set of frozen aqueous solutions of known mass and concentration of the persistent radical compound, 4-hydroxy-2,2,6,6-tetramethylpiperidine-*N*-oxyl (TEMPOL; molar mass 172.24 g mol^−1^).

### ^13^C NMR measurements

24 μL (4 × 6 μL) of sample was dissolved in an NMR tube containing 500 μL D_2_O doped with 2 mM gadolinium chloride. Room-temperature ^13^C NMR measurements were carried out using a 600 MHz spectrometer (Avance, Bruker, Billerica, MA, USA). All ^13^C NMR spectra were collected using an inverse gate ^13^C{^1^H}, 90° pulse, with a repetition time of 7 s, a spectral width of 210 ppm, and 1024 signal averages.

## Data availability

All relevant data has been included in the figures.

## Author contributions

J. S. L., A. P. G. and A. C designed the study. J. S. L. and A. P. G. performed the ESR and NMR measurements. J. S. L., A. P. G. and A. C. processed and analyzed the data. All authors participated in the writing of the manuscript. All authors have given approval to the final version of the manuscript.

## Conflicts of interest

A. C. was employed by General Electric Medical Systems Inc. at the time of manuscript preparation.

## Supplementary Material

SC-013-D2SC03038A-s001
