# Supplementary material for: Photochemistry of pyruvic acid is governed by photo-induced intermolecular electron transfer through hydrogen bonds
Source: Chem Sci. 2022 Oct 3;13(40):11849–55. doi: 10.1039/d2sc03038a (PMC9580485; doi:10.1039/d2sc03038a)
Supplement: SC-013-D2SC03038A-s001 [file SC-013-D2SC03038A-s001.pdf]

Electronic Supplementary Material (ESI) for Chemical Science.
This journal is © The Royal Society of Chemistry 2022

Supplementary Information

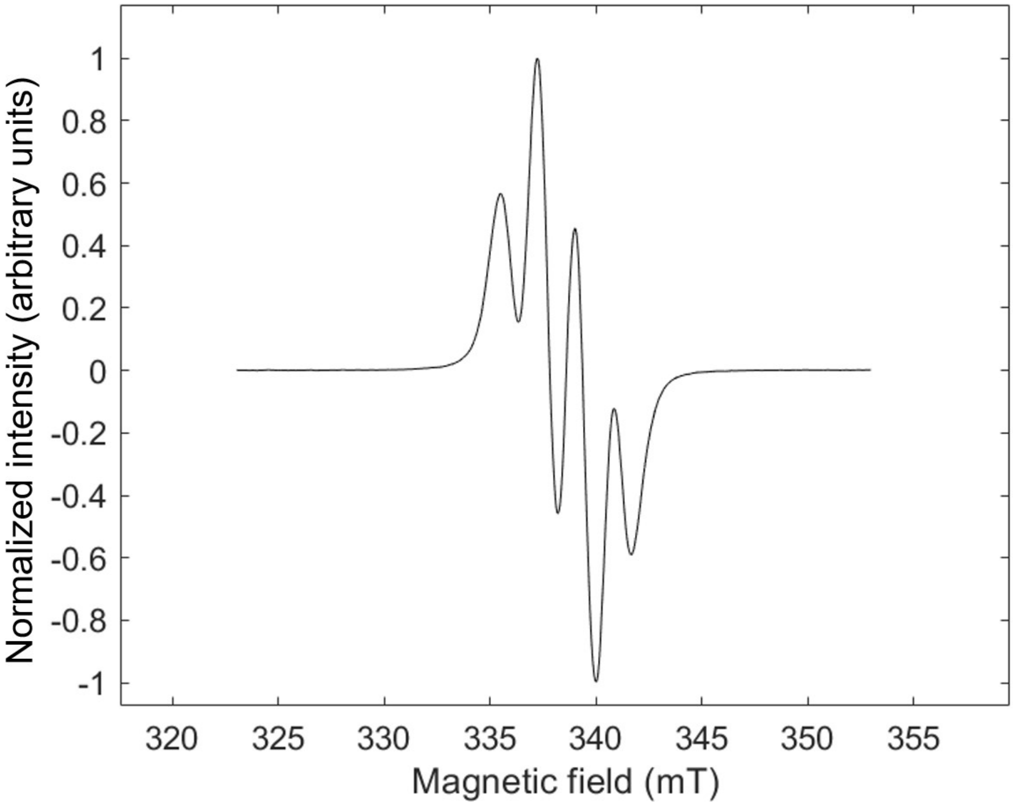

Fig. S1 X-band ESR spectrum measured at 77 K in photo-irradiated supercooled 300 mM aqueous pyruvic acid solution.

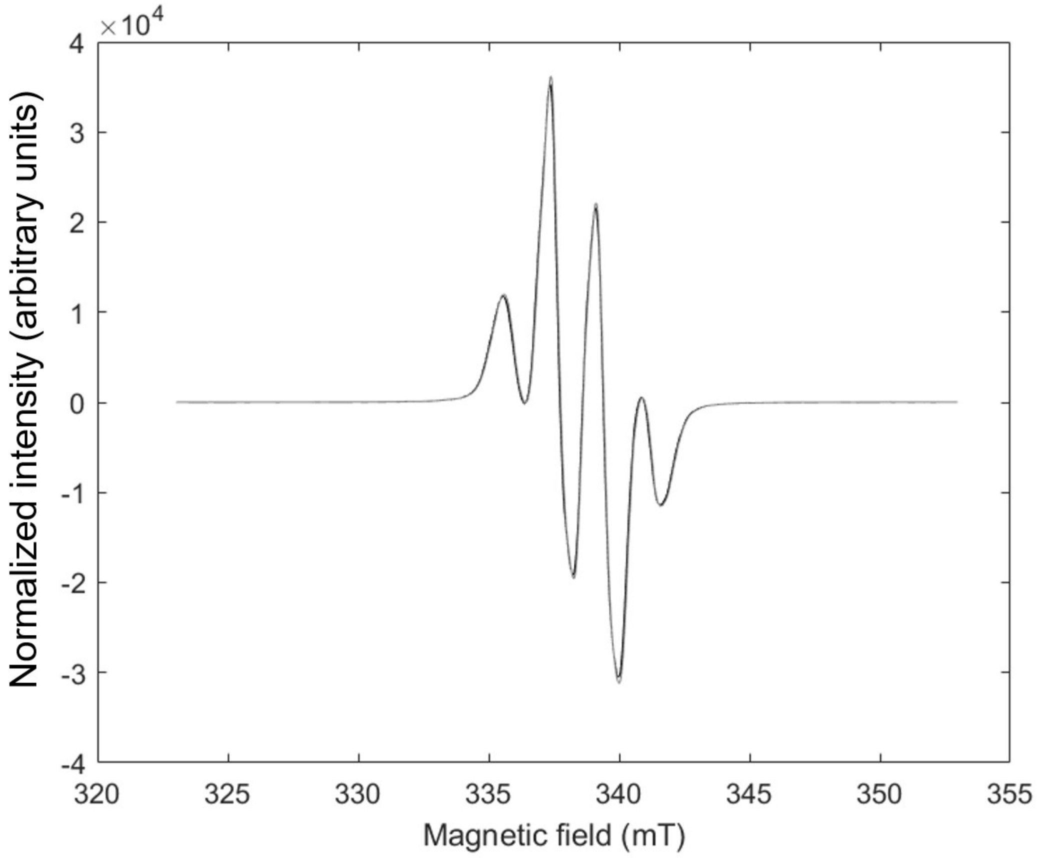

Fig. S2 X-band ESR spectra measured at 77 K in photo-irradiated supercooled neat pyruvic acid prepared with (black) and without (grey) sparging argon gas prior to snap freezing pyruvic acid. The radical concentration was 60±4 mM in both samples.

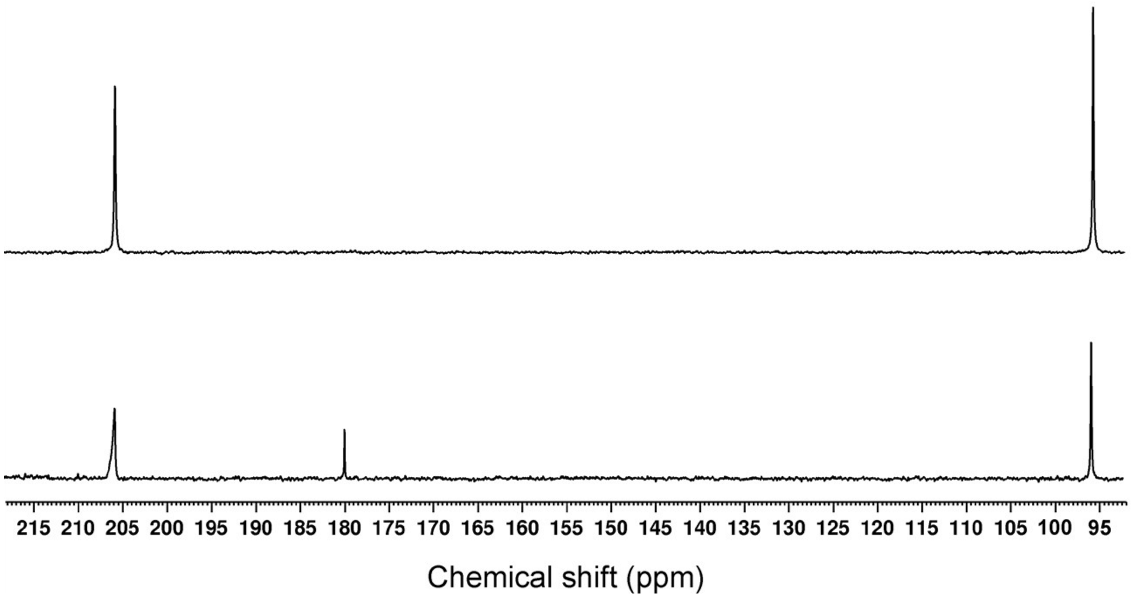

Fig. S3 Room-temperature liquid-state ^{13}C NMR spectra of dissolved photo-irradiated (bottom) and non-photo-irradiated (top) supercooled 300 mM aqueous [2-^{13}C]PA sample (24 μL) dissolved in 500 μL D_2O. The singlet peaks from the keto form (205.3 ppm) and the hydrated form (95.2 ppm) are visible. The singlet peak from the breakdown product [1-^{13}C]acetic acid appears on the photo-irradiated spectrum at 179.7 ppm.

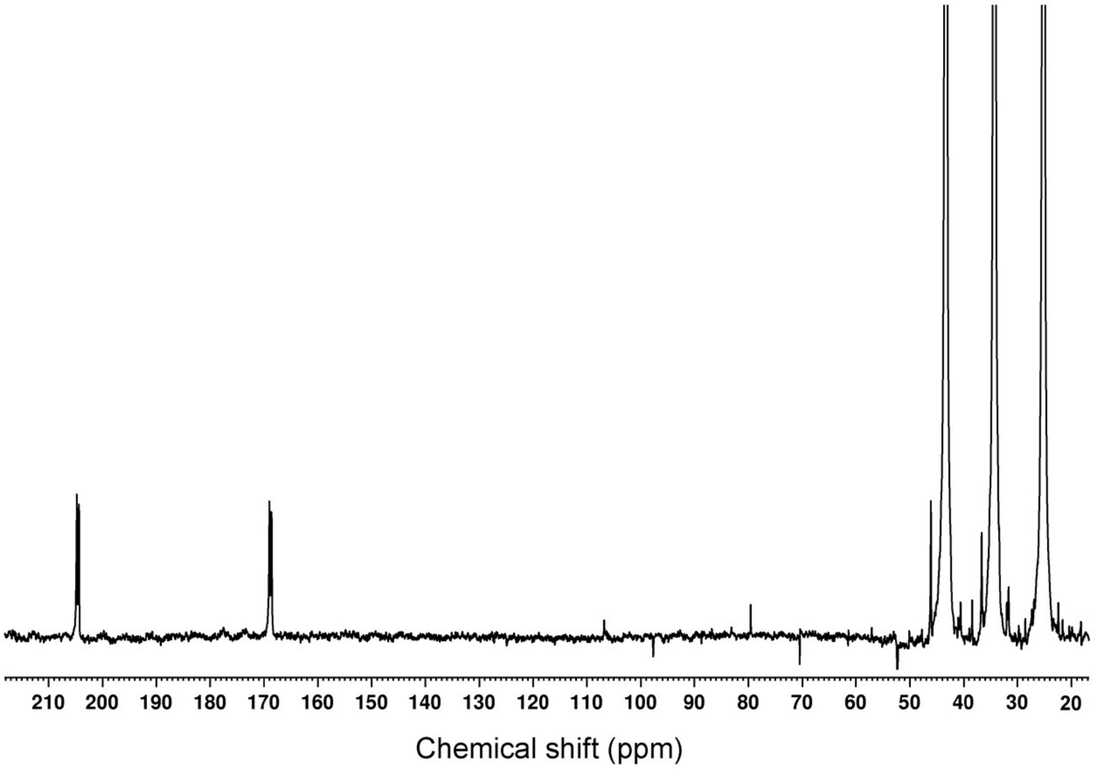

Fig. S4 Room-temperature liquid-state ^{13}C NMR spectrum (600 MHz) of photo-irradiated supercooled 300 mM [1,2-$^{13}C_2$]PA solution in hexane (24 µL) dissolved in 500 µL D_2O. The natural abundance ^{13}C hexane peaks (25 ppm, 34 ppm, and 43 ppm) were observed along with the keto form of [1,2-$^{13}C_2$]pyruvic acid (C1 at 169 ppm, and C2 at 205 ppm). No $^{13}CO_2$ was detected.
